# Influence of *HLA-DR* and -*DQ* alleles on autoantibody recognition of distinct epitopes within the juxtamembrane domain of the IA-2 autoantigen in type 1 diabetes

**DOI:** 10.1007/s00125-015-3803-5

**Published:** 2015-11-13

**Authors:** Carolyn C. Richardson, Kerry A. McLaughlin, Diana Morgan, Richard G. Feltbower, Michael R. Christie

**Affiliations:** Division of Diabetes & Nutritional Sciences, King’s College London Guy’s Campus, London, UK; School of Life Sciences, Joseph Banks Laboratories, University of Lincoln, Lincoln, LN6 7DL UK; Division of Epidemiology & Biostatistics, School of Medicine, University of Leeds, Leeds, UK

**Keywords:** Autoantibodies, Epitopes, *HLA-DQ*, *HLA-DR*, IA-2

## Abstract

**Aims/hypothesis:**

Insulinoma-associated protein 2 (IA-2) is a major target of autoimmunity in type 1 diabetes. When first detected, IA-2-autoantibodies commonly bind epitopes in the juxtamembrane (JM) domain of IA-2 and antibody responses subsequently spread to the tyrosine phosphatase domain. Definition of structures of epitopes in the JM domain, and genetic requirements for autoimmunity to these epitopes, is important for our understanding of initiation and progression of autoimmunity. The aims of this study were to investigate the contribution of individual amino acids in the IA-2 JM domain to antibody binding to these epitopes and the role of HLA genotypes in determining epitope specificity.

**Methods:**

Regions of the JM domain recognised by autoantibodies were identified by peptide competition and inhibitory effects of alanine substitutions of residues within the JM region. Antibody binding was determined by radioligand binding assays using sera from patients genotyped for *HLA-DRB1* and -*DQB1* alleles.

**Results:**

Patients were categorised into two distinct groups of JM antibody reactivity according to peptide inhibition. Inhibition by substitutions of individual amino acids within the JM domain differed between patients, indicating heterogeneity in epitope recognition. Cluster analysis defined six groups of residues having similar inhibitory effects on antibody binding, with three clusters showing differences in patients affected or unaffected by peptide. One cluster demonstrated significant differences in antibody binding between HLA-DRB1*04 and HLA-DRB1*07 patients and within DRB1*04 individuals; antibody recognition of a second cluster depended on expression of HLA-DQB1*0302.

**Conclusions/interpretation:**

The results identify amino acids contributing to distinct epitopes on IA-2, with both *HLA-DR* and *HLA-DQ* alleles influencing epitope specificity.

## Introduction

The strong association of HLA-DRB1*03, -DRB1*04 and linked *DQ* alleles with the development of type 1 diabetes is long established but the molecular mechanisms underlying HLA-mediated susceptibility are still obscure. It is widely accepted that HLA gene products mediate their effects via the presentation of peptides derived from islet autoantigens [1], and associations between expression of HLA alleles and the presence of antibodies to the autoantigens glutamate decarboxylase (HLA-DRB1*03) [2], insulin (HLA-DRB1*04) [3] and insulinoma-associated protein 2 (IA-2; HLA-DRB1*04, HLA-DRB1*07 and HLA-DRB1*09) [4, 5] support roles for HLA in regulating autoimmune responses to specific islet proteins. For the IA-2 autoantigen, relationships between HLA-DRB1*04 expression, the detection of T cell responses to specific IA-2 peptides and the presence of autoantibodies to specific regions of the antigen provide evidence of close links of HLA alleles with both T cell and B cell responses to a major autoantigen in type 1 diabetes [6]. B cell responses to IA-2 in the period before diabetes onset are progressive, with antibodies in the early phase of disease frequently recognising epitopes within the juxtamembrane (JM) domain of the protein, later spreading to epitopes in the protein tyrosine phosphatase (PTP) domain and to the closely related IA-2beta [7]. This diversification of the autoimmune response may be critical for disease progression [7]. Within the JM domain of IA-2 there are at least two distinct epitope regions, and B cell responses to these show different associations with HLA alleles [8]. The aim of this study was to fine-map epitopes for type 1 diabetes-associated autoantibodies within the JM domain of IA-2 by alanine scanning mutagenesis and to further explore HLA associations with antibody recognition of the epitope regions identified.

## Methods

### Participants

Blood samples were obtained from 140 type 1 diabetic patients recruited within 6 months of diagnosis of disease from clinics in West Yorkshire and King’s College Hospital, London, UK with informed consent and approval from the Yorkshire and the Humber – Bradford Leeds and the King’s College Hospital Research Ethics Committees for studies on the specificity of B cell and T cell responses in disease. Ethical approval for the study in Yorkshire restricted recruitment to patients ≥12 years of age, so there was an under-representation of young children. The mean age of patients was 18.8 years (range 8–36 years) and 94 (67%) were male. Blood samples were used for analysis of serum autoantibodies (see below) and for genotyping of *HLA-DRB1* and *-DQB1* loci by PCR amplification of genomic DNA using sequence-specific primers [9]. The autoantibody frequency and HLA genotypes expressed by the patients studied are shown in Table 1.

**Table 1:** Immune and HLA characteristics of the patient population

Variable	*n* (%)
Total number	140
GADA	114 (81%)
ZnT8A	82 (59%)
IA-2A	96 (69%)
IA-2-PTPA	78 (56%)
IA-2-JMA	51 (36%)
HLA-DRB1*03/DRB1*04	37 (26%)
HLA-DRB1*04/DRB1*04	12 (9%)
HLA-DRB1*04/X	35 (25%)
HLA-DRB1*03/DRB1*03	17 (12%)
HLA-DRB1*03/X	20 (14%)
*HLA-X/X*	19 (14%)

Numbers (*n*) and frequency (%) for positivity for islet autoantibodies and HLA genotypes are shown

IA-2A, antibodies to IA-2ic; IA-2-JMA, antibodies to IA-2 JM; IA-2-PTPA, antibodies to IA-2 PTP; X, non-HLA-DRB1*03 or DRB1*04

### Antibody analysis

Antibodies to glutamate decarboxylase (GADA), zinc transporter-8 (ZnT8A), and the intracytoplasmic (IA-2ic, residues 606-979) and PTP (694-979) domains of IA-2 were analysed by radioligand binding assay as previously described [10–12]. The sensitivity and specificity of the IA-2 antibody test in the 2012 Immunology of Diabetes Society Autoantibody workshop was 62% and 100%, respectively. Antibodies to the IA-2 JM domain (residues 606–700) were analysed using complementary DNA (cDNA) representing a chimeric construct in which residues 701–910 of the IA-2ic domain were replaced by residues 5–216 of human PTP1B, thereby deleting PTP domain epitopes. The cDNA for the IA-2 JM chimera was transcribed and translated in vitro in the presence of ^35^S-methionine (PerkinElmer, Coventry, UK) using the TNT Quick coupled transcription and translation system (Promega, Southampton, UK). Radiolabelled protein representing 20,000 cpm was incubated with test sera for 16 h at 4°C and a polyclonal rabbit antibody to IA-2 with predominant reactivity to the JM domain was included as a positive control. JM antibody units for each sample were calculated as a percentage of radioactivity immunoprecipitated by antibodies in the sample relative to that of the positive control. For peptide blocking studies, 5 μg of synthetic IA-2 peptides representing amino acids 601–620, 611–630 and 621–640 of IA-2 were added during the incubation. Immune complexes were captured on protein A Sepharose (Sigma, Poole, UK) and, after washing, the quantity of radiolabelled antigen bound was determined by scintillation counting. Peptides representing amino acids outside the 601–640 region did not block serum autoantibody binding to the IA-2 JM domain construct.

To evaluate the contribution of single amino acids within the JM domain to autoantibody binding, mutant IA-2 JM chimera constructs incorporating alanine substitutions at residues described in the text were generated using the QuikChange site-directed mutagenesis kit (Agilent Technologies, Stockport, UK) according to the manufacturer’s instructions. Successful incorporation of mutations was confirmed by sequencing (Source Bioscience, Nottingham, UK). Mutant constructs were transcribed and translated in vitro in the presence of ^35^S methionine and used together with wild-type constructs in radioligand binding assays as described above. The effect of individual amino acid substitutions on binding to autoantibodies in patients’ sera was calculated relative to that of the wild-type construct as per cent inhibition.

### Statistical analysis

Similarities in inhibitory effects of individual amino acid substitutions on binding of antibodies in type 1 diabetic patients to mutated IA-2 JM constructs were analysed by hierarchical cluster analysis by use of the furthest neighbour method using the Statistics Package for Social Sciences version 22 (SPSS; IBM, Portsmouth, UK). The significance of differences in inhibitory effects of individual or clusters of amino acid substitutions were analysed by two-way ANOVA with Sidak’s correction for multiple comparisons, or by Student’s *t-*test, as appropriate. ANOVA and *t-*tests were performed using Prism version 6 (GraphPad Software, La Jolla, CA, USA). Data are presented as means ± SEM.

## Results

### Characterisation of IA-2 JM domain autoantibodies in type 1 diabetes

The use of chimeric IA-2 constructs and competition studies using synthetic peptides has previously identified two distinct epitopes within the IA-2 JM domain contained within IA-2 regions 611–620 (JM1) and 621–630 (JM2) [8]. Substitutions of amino acids within the 619–631 region have revealed further heterogeneity in autoantibody recognition of the JM domain [13]. To define autoantibody epitopes within this region, sera from patients positive for antibodies to the IA-2 JM chimera were initially used in competition studies with synthetic 20-mer peptides. Alanine scanning mutagenesis was subsequently performed over the region 608–639 of the IA-2 JM domain.

**Fig. 1:**
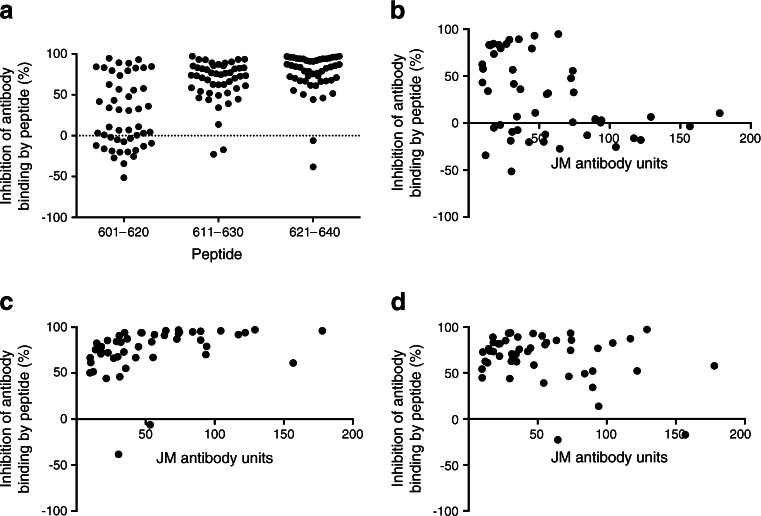
Scatter plots (**a**) showing inhibitory effects of synthetic 20-mer peptides representing amino acids 601–620, 611–630 and 621–640 on serum antibody binding to an IA-2 JM domain construct. Inhibition of individual patients’ antibodies is expressed as per cent inhibition of binding in absence of peptide. The relationships of inhibition by peptides 601–620 (**b**), 611–630 (**c**) and 621–640 (**d**) to levels of antibodies to the IA-2 JM domain construct are also shown

Of the 51 patients positive for antibodies to the IA-2 JM chimera in the initial screen, there was sufficient serum from 49 for detailed characterisation of antibody recognition of epitopes within the JM domain. These samples could be segregated into two groups on the basis of inhibition by a synthetic 20-mer peptide representing the 601–620 region of IA-2. Thus, 25 patients demonstrated ≤10% inhibition of antibody binding to the IA-2 JM construct by the peptide, with the remainder being inhibited by 30–95% (Fig. 1a). Samples inhibited by the 601–620 peptide had JM antibody levels of <80 units (Fig. 1b). This resistance to peptide inhibition was retained after serial dilution of those samples with high JM antibody levels to submaximal levels of antigen binding. The average inhibition by peptide of binding of antibodies in the eight sera with the highest JM antibody levels at neat, 1:2 and 1:4 dilutions were 1.26%, 4.91% and 0.26%, respectively. Antibody binding for almost all samples was inhibited by the 611–630 and 621–640 peptides (Fig. 1a), which suggests a crucial role of the 621–640 region of IA-2 in binding of all JM autoantibodies. Inhibition by 611–630 and 621–640 peptides was independent of JM antibody levels (Fig. 1c, d).

**Fig. 2:**
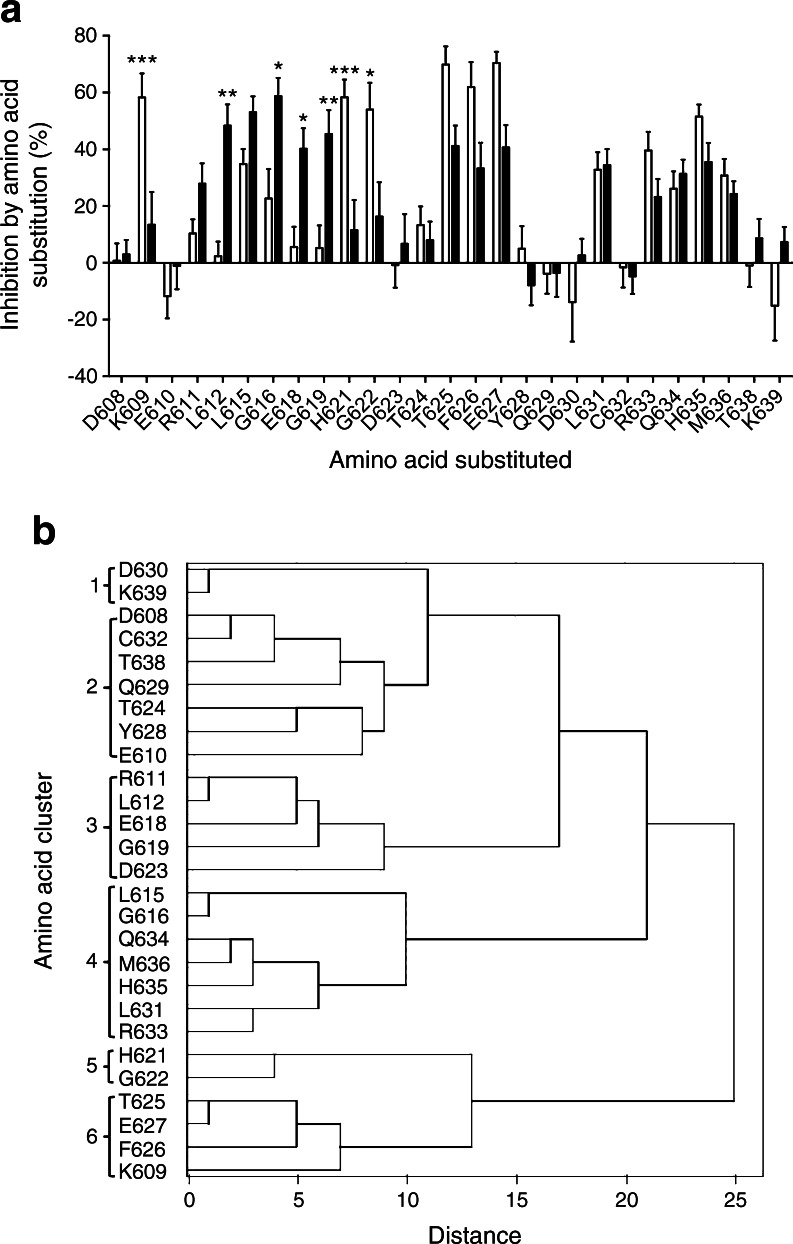
(**a**) Influence of substitutions of individual amino acids within the 608–639 region of IA-2 expressed in the IA-2 JM domain construct on binding of antibodies in sera from 49 JM antibody-positive patients grouped according to inhibitory effects (white bars, not inhibited; black bars, inhibited) of peptide 601–620. The significance of differences of effects of individual amino acid substitutions between groups affected or not affected by the 601–620 peptide is shown (*p < 0.05, **p < 0.01, ***p < 0.001). (**b**) Dendrogram illustrating results of cluster analysis of data from experiments evaluating similarities in inhibitory effects of individual amino acid substitutions. Residues with similar inhibitory effects are grouped into clusters defined by distance in the dendrogram being ≤10

To identify specific amino acids implicated in serum autoantibody recognition of JM domain epitopes, alanine substitutions of individual amino acids represented by the 608–639 region of IA-2 were made in the IA-2 JM chimeric construct. The influence of each substitution on binding of autoantibodies in sera from the 49 patients positive for antibodies to the IA-2 JM chimera was then evaluated. Clear differences were observed in the inhibitory effects of individual amino acid substitutions on antibody binding between samples that were inhibited or unaffected by the 601–620 peptide in the competition studies (Fig. 2a). Inhibition by individual alanine substitutions was largely independent of JM antibody levels in individual sera, with the exception of two residues in cluster 3, residues 611 and 612, where the highest inhibition was seen in low titre sera. Samples in which antibody binding was inhibited by the 601–620 peptide showed significantly higher mean inhibition by alanine substitutions of amino acids 612, 616, 618 and 619, whereas those unaffected by the peptide demonstrated greater inhibition by substitutions of amino acids 609, 621 and 622. Substitutions of amino acids 615, 631, 633, 634, 635 and 636 influenced antibody binding, but affected samples irrespective of inhibition by the 601–620 peptide in the competition study.

**Fig. 3:**
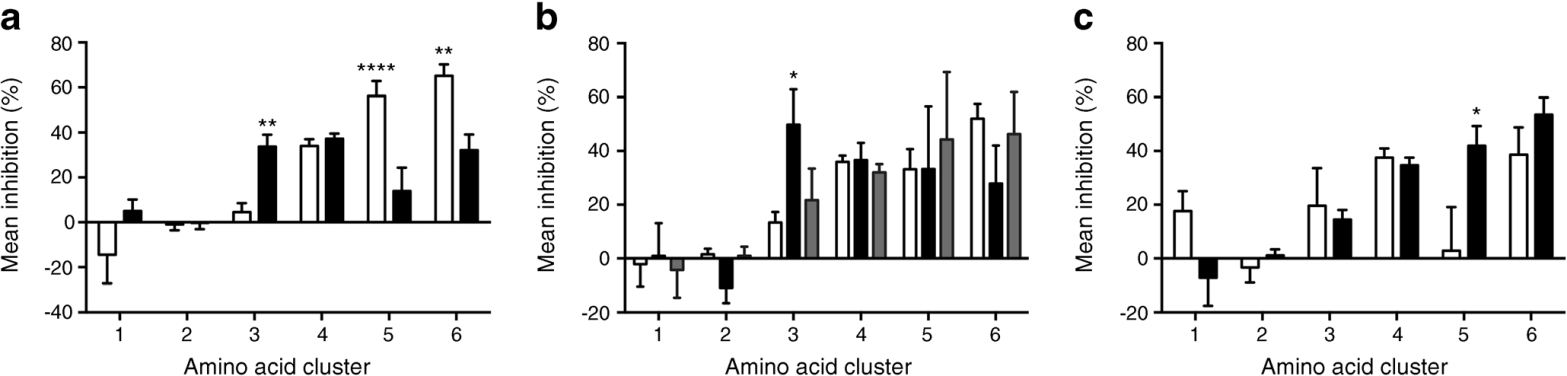
Mean per cent inhibition by alanine substitutions of residues within clusters 1–6 shown in Fig. 2b on the binding of antibodies in sera from 49 type 1 diabetic patients to the IA-2 JM construct. Patients were grouped according to (**a**) effects of peptide 601–620 on JM antibody binding (white bars, not inhibited; black bars, inhibited); (**b**) *HLA-DR* alleles (white bars, HLA-DRB1*04; black bars, HLA-DRB1*07; grey bars, other HLA alleles); or (**c**) *HLA-DQ* alleles within the HLA-DRB1*04 patients (white bars, HLA-DRB1*04-DQB1*0301; black bars, HLA-DRB1*04-DQB1*0302). The significance of differences of the mean inhibition by amino acid substitutions within each cluster between patients grouped according to peptide 601–620 inhibition (**a**) or HLA alleles expressed (**b**, **c**) is shown (*p < 0.05, **p < 0.01, ****p < 0.0001)

Similarities amongst individual amino acids in inhibitory effects on serum antibody binding after alanine substitution were investigated by hierarchical cluster analysis in order to identify groups of residues that may contribute to common antibody epitopes. The analysis identified six distinct clusters grouped with distance ≤10 in the dendrogram from the analysis (Fig. 2b). Samples categorised according to inhibitory effects of the 601–620 peptide showed different mean inhibition by substitutions of amino acids within each cluster. Substitutions in amino acids grouped as cluster 3 (residues 611, 612, 618, 619, 623) preferentially affected samples inhibited by the 601–620 peptide, whereas those in cluster 5 (621, 622) and cluster 6 (609, 625, 626, 627) showed strong inhibitory effects in those unaffected by the peptide (Fig. 3a). Substitutions of amino acids in cluster 4 (615, 616, 631, 633, 635, 636) affected most samples irrespective of peptide 601–620 inhibition, whereas those in cluster 1 (630 and 639) and cluster 2 (608, 610, 624, 628, 629, 632, 638) rarely affected antibody binding.

### Influence of HLA alleles on JM antibody binding

The mean inhibition of serum antibody binding by substitution of amino acids within each cluster shown in Fig. 2b was used to analyse the effects of *HLA-DR* and *HLA-DQ* alleles on autoantibody recognition of epitopes within the IA-2 JM domain. The majority of the 49 patients positive for IA-2-JM antibodies in the epitope analysis expressed either HLA-DRB1*04 (*n* = 37) or HLA-DRB1*07 (*n* = 7). Patients expressing HLA-DRB1*07 had significantly higher mean inhibition by substitution of cluster 3 amino acids (611, 612, 618, 619, 623) than those with HLA-DRB1*04 (Fig. 3b). Within HLA-DRB1*04 patients, significant differences in mean inhibition by residues 621 and 622 (cluster 5) were observed between those with HLA-DQB1*0302 and –DQB1*0301 alleles (Fig. 3c). Furthermore, patients with HLA-DRB1*03/DRB1*04, -DRB1*01/DRB1*04 or –DRB1*04/DRB1*13 had significantly lower mean inhibition by substitutions of cluster 3 residues (8.4 ± 4.7%, *n* = 26) than those with other HLA genotypes (30.9 ± 5.4%, *n* = 23; *p* < 0.005). The results demonstrate influences of both HLA-DR and DQ genes on autoimmune recognition of the IA-2 JM domain.

## Discussion

The first appearance of IA-2 autoimmunity in type 1 diabetes is often associated with the early detection of autoantibodies to the JM domain of the molecule. The association of the JM antibody response with HLA-DRB1*04, both in the period before and at the time of diabetes onset, in this study and other studies [6, 8] implicates immune responses to determinants within the JM domain as a potential contributor to diabetes susceptibility conferred by this allele. There is evidence of heterogeneity in autoantibody epitopes within the JM domain [13] and autoimmune responses to individual JM domain epitopes may have different associations with HLA genotypes [8]. In order to better understand the potential role of autoimmunity to the IA-2 JM domain in HLA-mediated disease susceptibility, we have undertaken a detailed analysis of the effects of individual amino acids within the IA-2 JM domain on autoantibody binding to identify clusters of amino acids with similar inhibitory effects of alanine substitution, implicit of common epitope regions. Antibody recognition of these epitope clusters is associated with different HLA-DR and DQ genotypes, consistent with a role for the products of these loci in the regulation of autoimmunity to the IA-2 JM region.

In agreement with previous observations of at least two distinct patterns of autoantibody binding to the IA-2 JM domain designated JM1 and JM2 [8], patients with type 1 diabetes in this study could be allocated to two groups according to inhibitory effects on antibody binding of a synthetic peptide representing the 601–620 region of the protein (Fig. 1a). However, alanine scanning mutagenesis revealed further heterogeneity between patients in amino acids affecting binding of antibodies, suggesting that the precise structure of epitopes in the JM1 and JM2 regions differed between patients. Groups of amino acids showing similarities in inhibitory effects of alanine substitution could be identified by cluster analysis, and these may represent common epitopes on the molecule recognised by subgroups of patients. The amino acid clusters could be categorised according to their general influence on antibody binding: (1) those residues where alanine substitution had minimal effects on antibody binding with most sera (clusters 1, 2); (2) residues where substitution affected antibody binding primarily in patients whose IA-2 JM antibodies were inhibited by the 601–620 peptide (cluster 3); (3) residues that contributed to antibody binding in patients with IA-2 JM antibodies unaffected by the 601–620 peptide (clusters 5, 6); and (4) residues where alanine substitution inhibited binding of antibodies in most sera, irrespective of effects of the 601–620 peptide (cluster 4). Lampasona et al [13] have previously analysed the effects of amino acid substitutions in the 619–631 region of IA-2 and we observed similar findings. Hence, inhibitory effects of alanine substitution of residue 619 (within cluster 3 in our study) defined a ‘JM1’ epitope in the Lampasona study and those of residues 621 and 622 (cluster 5) and 625, 626 and 627 (cluster 6) were found in all ‘JM2’ designated sera illustrated in the paper.

We have now extended the analysis of effects of amino acid substitutions across the entire JM epitope region. Our results demonstrate that mutations of amino acids that are widely separated in the linear IA-2 sequence affect binding of antibodies in individual patient sera and are contained within clusters of amino acids with similar inhibitory effects on antibody binding. It has previously been suggested that antibodies to the JM domain bind linear epitopes, on the basis that these can be represented by short sequences of JM amino acids contained within, for example, synthetic peptides [8]. Our new data suggest that, like other defined epitopes for antibodies in type 1 diabetes, protein conformation is also important for optimal binding of antibodies to the IA-2 JM domain, with noncontiguous amino acids contributing to the antibody epitopes. In particular, cluster analysis identified similar inhibitory effects of residues 609, 625, 626 and 627 (cluster 6) on JM antibody binding, with residue 609 being relatively far in the linear sequence of the JM domain from other contributing residues. This finding suggests either that alanine substitution of the lysine at residue 609 disturbs the conformation of the 625–627 region, for example by disrupting ionic interactions, or that the JM region is folded such that residue 609 is brought in close proximity to the 625–627 region and participates directly in antibody binding. Mouse monoclonal antibodies to the IA-2 JM domain having similar binding characteristics to those seen in human type 1 diabetes [14, 15], also displayed sensitivity to amino acid substitutions within noncontiguous regions of the JM domain [16]. Thus, four different mouse monoclonal antibodies to the IA-2 JM domain were all inhibited by alanine substitutions in the cluster 4 amino acids 615, 635 and 636, with each antibody being also affected by different amino acid substitutions elsewhere in the JM domain, similar to the observations with the patient sera. Together these results identify two distinct regions located at either end of the JM antibody-binding region each containing cluster 4 residues, one represented by amino acids 615 and 616 and the other by residues 631, 633, 634, 635 and 636, that have general importance for autoantibody binding, with the characteristic epitope for the antibody being defined by more specific inhibitory effects of substitutions of amino acids within clusters 3, 5 or 6. The epitope represented by amino acids in cluster 3 is dominant in sera inhibited by the 601–620 peptide (defined as JM1 epitope in [8]) and patients with antibodies to this epitope tend to have lower JM antibody levels, as seen for antibodies inhibited by the 601–620 peptide (Fig. 1b) and those affected by alanine substitution of the cluster 3 residues 611 and 612.

Our data also suggest that autoantibody reactivity to specific epitopes represented by amino acids within clusters 3 and 5 may be influenced by HLA (Fig. 3b, c). It has been demonstrated previously that autoantibody responses to the IA-2 JM domain are associated with expression of HLA-DRB1*04 [8], with autoantibody frequencies being similar in DRB1*04-DQB1*0301 and DRB1*04-DQB1*0302 individuals, suggesting a primary association with the DR, rather than DQ, locus [6]. However, dissection of the JM autoantibody response to individual epitopes reveals a more complex influence of the HLA region on the specificity of autoimmunity to the JM domain, with involvement of both *HLA-DR* and *HLA-DQ* alleles. Substitution of amino acids within cluster 3 produced significantly higher inhibition of antibody binding in patients expressing HLA-DRB1*07. HLA-DRB1*07 has different effects on disease susceptibility depending on the *HLA-DQA* genotypes expressed [17]. A positive association of HLA-DRB1*07 with IA-2 autoantibodies has previously been reported and the authors suggested that this association may be secondary to effects of *HLA-DQA1* alleles [5]. In that study, JM antibodies were negatively associated with HLA-DRB1*07, although the analysis used a chimeric construct missing region 633–636 that, according to the results of our study, may be required for optimal JM antibody binding. The two studies are, nevertheless, consistent with an influence of HLA-DRB1*07 or linked *HLA-DQ* gene products on the IA-2 autoimmune response. Patients with HLA-DRB1*03/DRB1*04, DRB1*01/DRB1*04 or DRB1*04/DRB1*13 had significantly lower inhibition by cluster 3 substitutions than other HLA genotypes. This observation is consistent with the findings of Bearzatto et al [8], who found that antibodies to the JM2 epitope in the absence of those to JM1 (cluster 3 in our study) were found in relatives of individuals with type 1 diabetes with HLA-DRB1*03/DRB1*04, DRB1*01/DRB1*04 or DRB1*04/DRB1*13 genotypes. Furthermore, inhibitory effects of cluster 5 substitutions on JM antibody binding were associated with HLA-DQB1*0302 expression within HLA-DRB1*04 patients, suggesting that this major type 1 diabetes susceptibility allele may influence the specificity of the IA-2 autoimmune response, in particular to the JM region that may dominate around the time of first appearance of IA-2 autoimmunity. This association is almost certainly secondary to a primary association of DQ8-restricted T cell responses to as yet undefined T cell determinants. Candidates may include peptides within the region 601–633 that have been shown to specifically stimulate T cell responses in HLA-DQB1*0302 transgenic mice [18].

The results of our study define epitopes for autoantibodies in a region of an autoantigen known to be important in the early phases of autoimmunity in type 1 diabetes and demonstrate an influence of both *HLA-DR* and -*DQ* alleles on the specificity of B cell responses to the IA-2 JM region. Autoantibody responses to the JM domain are primarily associated with the expression of HLA-DRB1*04, but antibodies in patients expressing HLA-DRB1*07 recognise a specific epitope represented by amino acids 611, 612, 618, 619 and 623. Furthermore, in HLA-DRB1*04 patients, antibody recognition of epitopes defined by residues 621 and 622 is impaired if HLA-DQB1*0301, rather than HLA-DQB1*0302, is expressed. The data illustrate the importance of dissecting the autoantibody response to individual epitopes on autoantigens to reveal responses influenced by HLA haplotypes expressed by the diabetic patient. Identification of T cell determinants with appropriate HLA restriction that are associated with responses to JM epitopes defined in this study will help to understand the molecular basis of HLA-mediated susceptibility to type 1 diabetes.

## Abbreviations

cDNA: Complementary DNA
GADA: Antibodies to glutamate decarboxylase
IA-2ic: Intracytoplasmic domain of insulinoma-associated protein 2
JM: Juxtamembrane
PTP: Protein tyrosine phosphatase
ZnT8A: Antibodies to zinc transporter-8

## References

[1] Todd JA (2010). Etiology of type 1 diabetes. Immunity.

[2] Knip M, Kukko M, Kulmala P (2002). Humoral beta-cell autoimmunity in relation to HLA-defined disease susceptibility in preclinical and clinical type 1 diabetes. Am J Med Genet.

[3] Ziegler R, Alper CA, Awdeh ZL (1991). Specific association of HLA-DR4 with increased prevalence and level of insulin autoantibodies in first-degree relatives of patients with type I diabetes. Diabetes.

[4] Genovese S, Bonfanti R, Bazzigaluppi E (1996). Association of IA-2 autoantibodies with HLA DR4 phenotypes in IDDM. Diabetologia.

[5] Williams AJK, Aitken RJ, Chandler MA-M, Gillespie KM, Lampasona V, Bingley PJ (2008). Autoantibodies to islet antigen-2 are associated with HLA-DRB1*07 and DRB1*09 haplotypes as well as DRB1*04 at onset of type 1 diabetes: the possible role of HLA-DQA in autoimmunity to IA-2. Diabetologia.

[6] McLaughlin KA, Gulati K, Richardson CC (2014). HLA-DR4-associated T and B cell responses to specific determinants on the IA-2 autoantigen in type 1 diabetes. J Immunol.

[7] Naserke HE, Ziegler AG, Lampasona V, Bonifacio E (1998). Early development and spreading of autoantibodies to epitopes of IA-2 and their association with progression to type 1 diabetes. J Immunol.

[8] Bearzatto M, Naserke H, Piquer S (2002). Two distinctly HLA-associated contiguous linear epitopes uniquely expressed within the islet antigen 2 molecule are major autoantibody epitopes of the diabetes-specific tyrosine phosphatase-like protein autoantigens. J Immunol.

[9] Bunce M, O’Neill CM, Barnardo MC (1995). Phototyping: comprehensive DNA typing for HLA-A, B, C, DRB1, DRB3, DRB4, DRB5 & DQB1 by PCR with 144 primer mixes utilizing sequence-specific primers (PCR-SSP). Tissue Antigens.

[10] Christie MR, Roll U, Payton MA, Hatfield ECI, Ziegler AG (1997). Validity of screening for individuals at risk for type 1 diabetes by combined analysis of antibodies to recombinant proteins. Diabetes Care.

[11] Hatfield ECI, Hawkes CJ, Payton MA, Christie MR (1997). Cross reactivity between IA-2 and phogrin/IA-2beta in binding of autoantibodies in IDDM. Diabetologia.

[12] Wenzlau JM, Juhl K, Yu L (2007). The cation efflux transporter ZnT8 (Slc30A8) is a major autoantigen in human type 1 diabetes. Proc Natl Acad Sci.

[13] Lampasona V, Belloni C, Piquer S, Bonicchio S, Furlan R, Bonifacio E (2008). Radiobinding assay for detecting autoantibodies to single epitopes. J Immunol Methods.

[14] Piquer S, Valera L, Lampasona V (2006). Monoclonal antibody 76F distinguishes IA-2 from IA-2beta and overlaps an autoantibody epitope. J Autoimmun.

[15] Ananieva-Jordanova R, Evans M, Nakamatsu T (2005). Isolation and characterisation of a human monoclonal autoantibody to the islet cell autoantigen IA-2. J Autoimmun.

[16] McLaughlin KA, Richardson CC, Williams S (2015). Relationships between major epitopes of the IA-2 autoantigen in type 1 diabetes: implications for determinant spreading. Clin Immunol.

[17] Noble JA, Johnson J, Lane JA, Valdes AM (2012). Race-specific type 1 diabetes risk of HLA-DR7 haplotypes. Tissue Antigens.

[18] Kudva YC, Deng Y-J, Godvindarajan R (2001). HLA-DQ8 transgenic and NOD mice recognize different epitopes within the cytoplasmic domain of the tyrosine phosphatase-like molecule, IA-2. Hum Immunol.

[19] Johnson CC, Mclaughlin KA, Morgan D, Feltbower RG, Christie MR (2014). Fine mapping of epitopes for antibodies to the juxtamembrane domain of IA-2 in type 1 diabetes. Diabetes.

